# A Complex Diagnosis of Ampullary Adenocarcinoma Presenting As Decompensated Cirrhosis

**DOI:** 10.7759/cureus.37566

**Published:** 2023-04-14

**Authors:** Humzah Iqbal, Arpine Petrosyan, Jennifer Yoon, Marina Roytman

**Affiliations:** 1 Internal Medicine, University of California, San Francisco, Fresno, USA; 2 Gastroenterology and Hepatology, University of California, San Francisco, Fresno, USA

**Keywords:** diagnostic and therapeutic ercp, endoscopic ultrasound (eus), liver and gall bladder disease, decompensated cirrhosis, ampullary carcinoma

## Abstract

Neoplasms arising from the ampulla of Vater are exceedingly rare, and there is a paucity of literature regarding their diagnosis and management. Ampullary cancer typically presents with jaundice and signs of biliary obstruction. We present a case of ampullary adenocarcinoma with concomitant choledocholithiasis that proved complex and diagnostically challenging.

## Introduction

Ampullary adenocarcinoma is a rare malignancy arising from the ampulla of Vater that accounts for only 0.2% of all gastrointestinal cancers [1]. There are two distinct histological subtypes of ampullary adenocarcinoma, intestinal and pancreaticobiliary. The distinction is based on whether the neoplasm originates from the intestinal epithelium or the distal ductal system [2]. Patients often present with obstructive jaundice, a presentation commonly seen with choledocholithiasis [3]. Given the rarity of the disease, diagnosing ampullary malignancy can be difficult when there is concurrent gallstone disease. Treatment often involves resection or chemoradiation; however, there is a paucity of literature regarding diagnosing and managing ampullary cancer [1]. We present a case of ampullary adenocarcinoma with a unique presentation and prolonged time to diagnosis.

This case was presented as a poster abstract at the Northern California Society for Clinical Gastroenterology (NCSCG) Liver Symposium in January 2023. 

## Case presentation

A 74-year-old man with prior alcohol use disorder presented to an outside hospital with a two-month history of progressive jaundice, generalized pruritus, and weight loss in October 2020. Initial labs are summarized in Table 1 and demonstrated a cholestatic pattern with significant elevations in total bilirubin and alkaline phosphatase.

**Table 1:** Lab values at initial presentation ALT: Alanine aminotransferase; AST: Aspartate transaminase; INR: International normalized ratio

Laboratory studies (reference range)	Value at initial presentation
Total bilirubin (0.3-12 mg/dL)	18 mg/dL
Alkaline phosphatase (25-100 U/L)	305 U/L
ALT (8-40 U/L)	59 U/L
AST (8-40 U/L)	75 U/L
INR (0.8-1.1)	1.0
Albumin (3.4-4.8 g/dL)	3.4 g/dL

**Figure 1:**
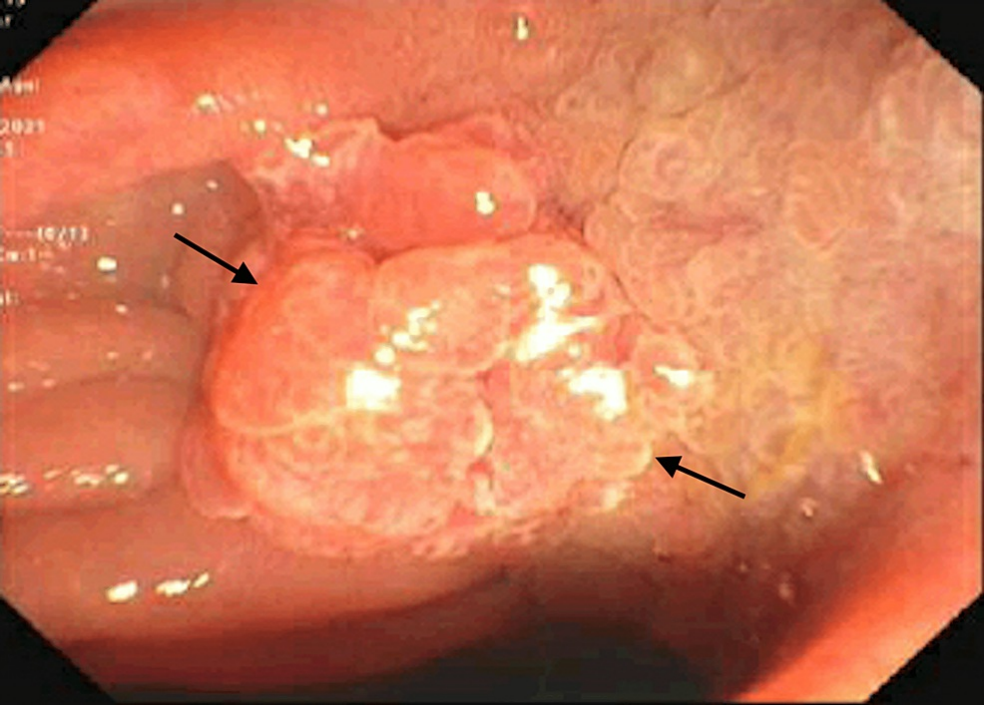
Outpatient ERCP demonstrating ampullary mass, which was biopsied and was negative for dysplasia or malignancy. ERCP: Endoscopic retrograde cholangiopancreatography

Previous outpatient work-up included an abdominal ultrasound, magnetic resonance cholangiopancreatography (MRCP), and endoscopic ultrasound (EUS), which demonstrated cholelithiasis, bile duct sludge, and dilation of the common bile duct (CBD) and pancreatic duct. Outpatient endoscopic retrograde cholangiopancreatography (ERCP) one week before admission showed CBD dilation and prominent major papilla (Figure 1). The biliary tree was swept with clearance of sludge; ampullary biopsy and sphincterotomy were performed. The biopsy was negative for dysplasia or malignancy.

During admission, the patient underwent repeat ERCP due to worsening hyperbilirubinemia which showed CBD dilation. A biliary stent was placed and subsequently removed (at a later time). The patient was discharged after demonstrating a downward trend of total bilirubin from 20 mg/dL to 11.4 mg/dL. A few weeks later, he was admitted to a different outside hospital for worsening abdominal distention, intermittent right upper quadrant pain, and overt hepatic encephalopathy. Liver tests were again elevated in a cholestatic pattern. He was diagnosed with decompensated cirrhosis, suspected due to prior alcohol use, and started on treatment for ascites and hepatic encephalopathy. He was discharged and referred to our outpatient hepatology clinic.

**Figure 2:**
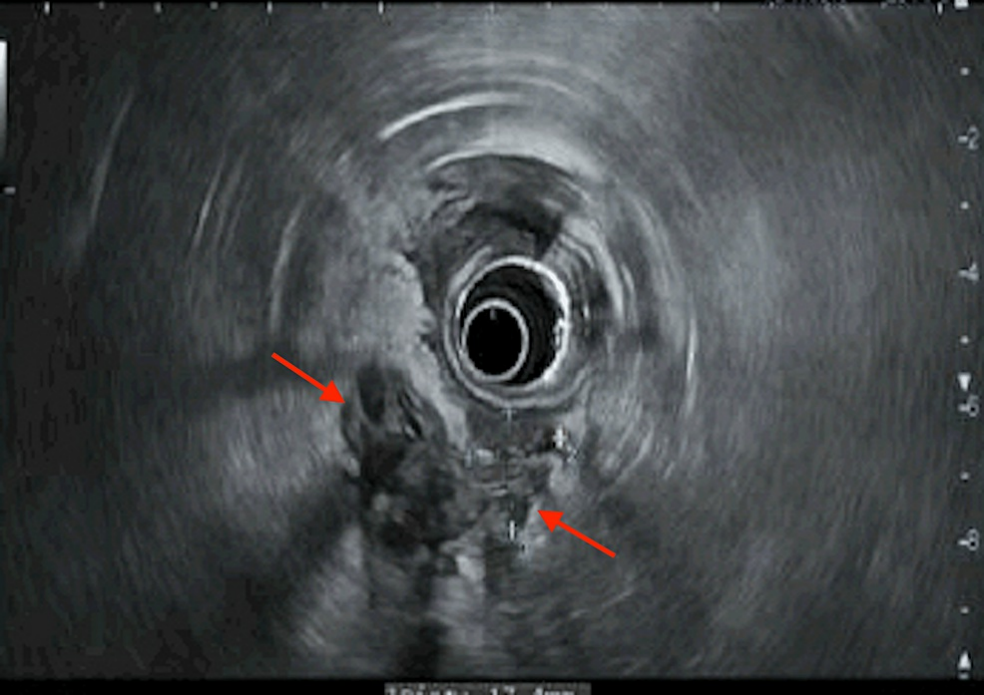
Patient's third EUS demonstrating ampullary mass, which was biopsied and showed adenocarcinoma. EUS: Endoscopic ultrasound

**Figure 3:**
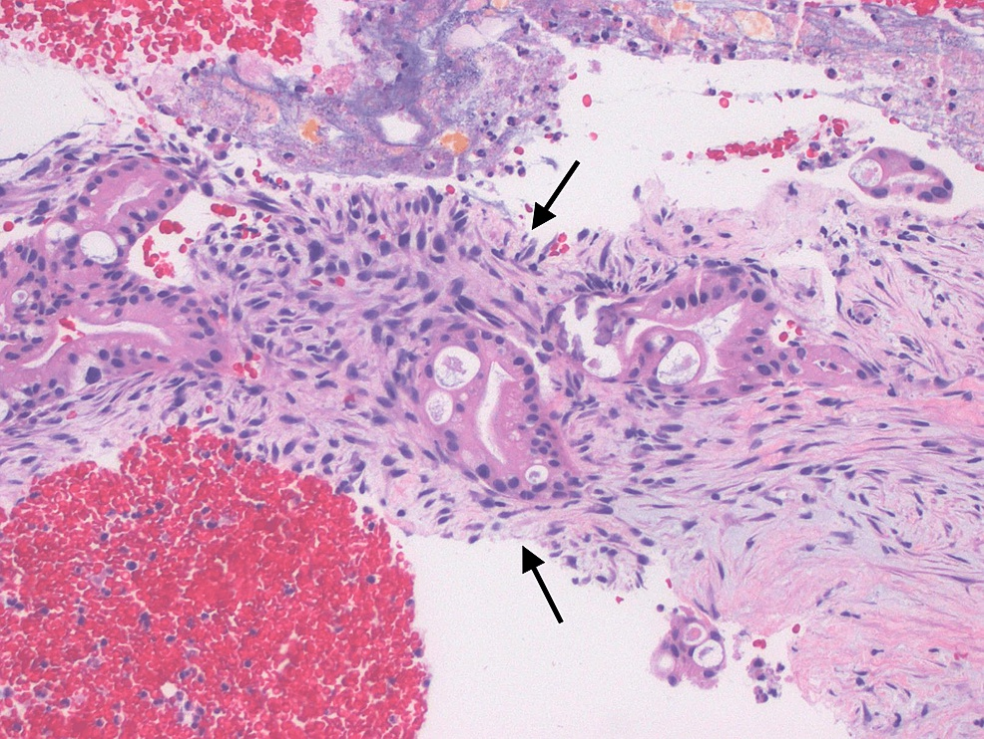
H&E stained slide of ampullary mass biopsy revealing invasive adenocarcinoma. H&E: Hematoxylin and eosin

**Figure 4:**
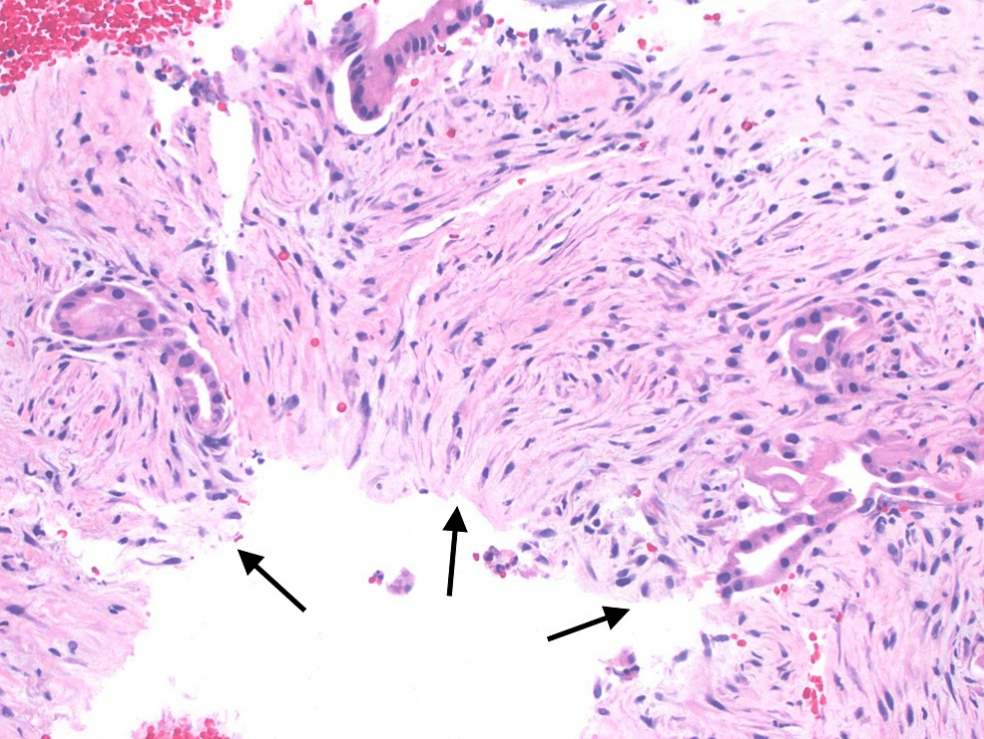
H&E stained slide of ampullary mass biopsy revealing invasive adenocarcinoma. H&E: Hematoxylin and eosin

Upon evaluation in our clinic, the differential diagnosis included ampullary dysfunction, cholangiocarcinoma, and stricture as a sequela of an impacted stone potentially in the setting of previously well-compensated alcoholic liver disease. The patient's case was presented to our multidisciplinary tumor board with the recommendation of repeating EUS. EUS with ampullary biopsy was performed in January 2021 and was again negative for dysplasia or malignancy. Notably, by this time, the patient's obstructive jaundice, ascites, and hepatic encephalopathy had resolved entirely, leading the clinicians to suspect that the patient's symptoms of decompensation were related to secondary biliary cirrhosis. He underwent laparoscopic cholecystectomy in May of 2021 without evidence of hepatic decompensation. He was seen in our hepatology clinic in August of 2021 with a recurrence of jaundice and pruritus. Repeat EUS (patient's 3rd) demonstrated an ampullary mass (Figure 2), and the biopsy revealed ampullary adenocarcinoma (Figure 3, 4) nearly 18 months after the initial presentation.

His disease was not considered resectable, and he was started on neoadjuvant chemotherapy with gemcitabine and paclitaxel. The patient did not tolerate chemotherapy and was subsequently transitioned to hospice care.

## Discussion

Common presentations of ampullary cancer include jaundice, abdominal pain, and pancreatitis [4]. Our patient presented with signs and symptoms of obstructive jaundice and was found to have an ampullary mass after extensive evaluation. Laboratory work-up revealing elevated total bilirubin and alkaline phosphatase with only mild elevations in transaminases were concerning for cholestasis; however, the patient's intermittent symptoms were more consistent with gallstone disease than an obstructing mass, which would present with more persistent symptoms [5]. The waxing and waning presentation of this case, the presence of gallstone disease, the seeming resolution of symptoms after several therapeutic interventions, and multiple negative ampullary biopsies confounded the final diagnosis of ampullary adenocarcinoma and ultimately delayed the diagnosis. Additionally, the patient had two distinct and unique presentations for ampullary adenocarcinoma, one of waxing and waning obstructive jaundice over 18 months and the other as decompensated cirrhosis. 

The patient likely had undiagnosed, long-standing, well-compensated cirrhosis or at least advanced fibrosis with the biliary obstruction contributing to secondary biliary cirrhosis. Rat models have demonstrated the development of cirrhosis after about 15 days of biliary obstruction with cholestasis [6]. A prospective cohort study by Negi et al. also found that the duration of biliary obstruction in humans was an independent predictor of the development of advanced hepatic fibrosis and secondary biliary cirrhosis [7]. The histopathologic appearance of our patient's ampullary mass may have contributed to the multiple negative biopsies, as his lesion was particularly well-differentiated. The presence ultimately diagnosed mild nuclear atypia, architectural complexity, and a non-lobular infiltrative pattern with desmoplastic fibrosis (Figure 3,4). Our patient was found to have the pancreaticobiliary subtype of ampullary adenocarcinoma, which is thought to have a poorer prognosis than the intestinal subtype. However, as many as 40% of cases are indistinguishable and considered "mixed" [8].

Ampullary adenocarcinoma generally has a favorable prognosis, with over 50% of patients undergoing surgical resection via pancreaticoduodenectomy [9]. However, in some cases, the disease may be unresectable and systemic chemotherapy may need to be initiated, as seen in our patient. Studies have demonstrated mixed results, some showing improved survival benefits and others no improvement with systemic chemotherapy without resection [10,11]. ESPAC-3, a phase III randomized control trial, found a 5-fluorouracil/leucovorin combination and gemcitabine monotherapy to have an increased median survival time. Both regimens are viable options for treating ampullary cancer [10].

Additionally, a retrospective study by Sideras et al. found that tumor cells in ampullary cancer express high levels of immune inhibitory molecules such as PD-L1, Gal-9, HVEM, and HLA-G, which can serve as potential targets for immunotherapy [12]. Interestingly, B7-H3 is a potential immunotherapeutic target in the pancreato-biliary subtype of ampullary adenocarcinoma, as it is frequently expressed in the cancer cells and the tumoral stroma [13]. Further studies are needed to elucidate the most effective management of ampullary cancer.

## Conclusions

Clinicians should have a high suspicion of ampullary malignancy in patients with decompensated cirrhosis presenting with obstructive jaundice to initiate appropriate and timely management. Diagnosis can be difficult when there is concurrent gallstone disease; clinicians should be aware of potential confounders. Patients generally have a favorable prognosis when detected early; however, delayed time to diagnosis can lead to increased morbidity and mortality.
